# A large-scale RNAi screen reveals that mitochondrial function is important for meiotic chromosome organization in oocytes

**DOI:** 10.1007/s00412-023-00784-9

**Published:** 2023-01-17

**Authors:** Karen Jule Nieken, Kathryn O’Brien, Alexander McDonnell, Liudmila Zhaunova, Hiroyuki Ohkura

**Affiliations:** Wellcome Centre for Cell Biology, School of Biological Sciences, University of Edinburgh, Edinburgh, EH9 3BF UK

**Keywords:** Drosophila, Meiosis, Oocytes, Chromatin, Mitochondria

## Abstract

In prophase of the first meiotic division, chromatin forms a compact spherical cluster called the karyosome within the enlarged oocyte nucleus in *Drosophila melanogaster*. Similar clustering of chromatin has been widely observed in oocytes in many species including humans. It was previously shown that the proper karyosome formation is required for faithful chromosome segregation, but knowledge about its formation and maintenance is limited. To identify genes involved in karyosome formation, we carried out a large-scale cytological screen using *Drosophila melanogaster* oocytes. This screen comprised 3916 genes expressed in ovaries, of which 106 genes triggered reproducible karyosome defects upon knockdown. The karyosome defects in 24 out of these 106 genes resulted from activation of the meiotic recombination checkpoint, suggesting possible roles in DNA repair or piRNA processing. The other genes identified in this screen include genes with functions linked to chromatin, nuclear envelope, and actin. We also found that silencing of genes with mitochondrial functions, including electron transport chain components, induced a distinct karyosome defect typically with de-clustered chromosomes located close to the nuclear envelope. Furthermore, mitochondrial dysfunction not only impairs karyosome formation and maintenance, but also delays synaptonemal complex disassembly in cells not destined to become the oocyte. These karyosome defects do not appear to be mediated by apoptosis. This large-scale unbiased study uncovered a set of genes required for karyosome formation and revealed a new link between mitochondrial dysfunction and chromatin organization in oocytes.

**Supplementary Information:**

The online version contains supplementary material available at 10.1007/s00412-023-00784-9.

## Introduction

Accurate transmission of genetic material in the germline is of vital importance to the offspring since the entire organism is affected. It is therefore critical that chromosomes are faithfully distributed during the formation of gametes in a specialized form of cell division known as meiosis. Errors in meiotic cell divisions are a frequent cause of infertility, miscarriages, and birth defects in humans. During these divisions, the meiotic chromatin undergoes dynamic rearrangements that remain to be understood at the molecular level.

During meiotic prophase in oocytes, clustering of meiotic chromosomes before the nuclear envelope breakdown is widely observed in many species including humans (Gruzova and Parfenov 1993). These structures are called the karyosphere or karyosome. The precise organization can be variable between species and also between developmental stages of oocytes in the same species (Gruzova and Parfenov 1993). The formation of the karyosphere/karyosome is often temporally coordinated with global repression of transcription in vivo (Bouniol-Baly et al. 1999). In mouse oocytes, clustering of meiotic chromosomes in the oocyte nucleus is correlated with developmental competency after fertilisation (Zuccotti et al. 1998, 2002). It has also been proposed that holding meiotic chromosomes together is important for assembling one meiotic spindle shared by all the chromosomes within the large volume of the oocyte without centrosomes (Cullen et al. 2005). Despite conservation and the importance of the karyosphere/karyosome, the molecular mechanism and regulation is not well understood.

In *Drosophila melanogaster* oocytes, the meiotic chromosomes form a compact spherical cluster called the karyosome in prophase of the first meiotic division. It was previously shown that karyosome formation is required for faithful chromosome segregation in *Drosophila* oocytes (Cullen et al. 2005), but knowledge about its formation and maintenance at the molecular level is limited. We established that proper karyosome formation requires the detachment of chromatin from the nuclear envelope (Lancaster et al. 2007). This detachment is mediated by phosphorylation of barrier-to-autointegration factor (BAF), a linker between chromatin and the nuclear envelope, by the conserved kinase NHK-1/Vrk (Lancaster et al. 2007). In addition, interaction between chromatin and the nuclear pores needs to be disrupted (Breuer and Ohkura 2015). Disruption of the interaction between chromatin and the nucleoporin Nup155 depends on other nucleoporins, Nup62 and Nup93 (Breuer and Ohkura 2015). Furthermore, the histone demethylase Kdm5/Lid is important for the spherical karyosome morphology, although its enzymatic activity is not required (Zhaunova et al. 2016). A mutation in the Src tyrosine kinase Src64, which is known to regulate actin dynamics, also disrupts the spherical karyosome morphology and decreases monomeric actin in the nucleus (Djagaeva et al. 2005), suggesting involvement of actin in karyosome formation. The conserved SR protein kinase (SRPK) is also essential for karyosome formation in early oogenesis as well as spindle microtubule assembly in mature oocytes (Loh et al. 2012). The substrates of SRPK mediating either of the processes have not been identified. Moreover, another protein called Encore is important for karyosome formation. It has been shown to interact and cooperate with the SCF ubiquitin ligase and the proteasome protein degradation machinery (Ohlmeyer and Schüpbach 2003). In summary, karyosome formation appears to be regulated by a multitude of genes involved in different pathways. We therefore expect further players to be involved in the intricate network that mediates karyosome formation and maintenance.

Karyosome formation is also impaired upon the meiotic recombination checkpoint (Ghabrial and Schüpbach 1999; González-Reyes et al. 1997; Styhler et al. 1998). DNA double-strand breaks (DSBs) are formed and repaired during recombination before karyosome formation. However, when DSBs are not repaired or continuously generated by uncontrolled germline retrotransposition, the meiotic checkpoint is activated and consequently interferes with proper karyosome formation (Chen et al. 2007; Klattenhoff et al. 2007; Mehrotra and McKim 2006; Pane et al. 2007). NHK-1 kinase, required for karyosome formation, has been identified as a crucial target suppressed by the meiotic checkpoint (Lancaster et al. 2010).

To gain an insight into how the karyosome formation is regulated at the molecular level, we carried out a large-scale screen and identified 106 genes important for proper karyosome formation. They encode both novel and known regulators of chromatin architecture, nuclear envelope structure, and the actin cytoskeleton. The karyosome defects caused by silencing of 24 of these genes are dependent on the meiotic recombination checkpoint, suggesting their roles in DSB repair or piRNA processing. Unexpectedly, genes with mitochondrial function have been identified, but the karyosome defects caused by their silencing are not mediated by apoptosis, which is known to link mitochondria to nuclear events.

## Results

### A large-scale screen identified 106 genes required for the integrity of the karyosome

**Fig. 1:**
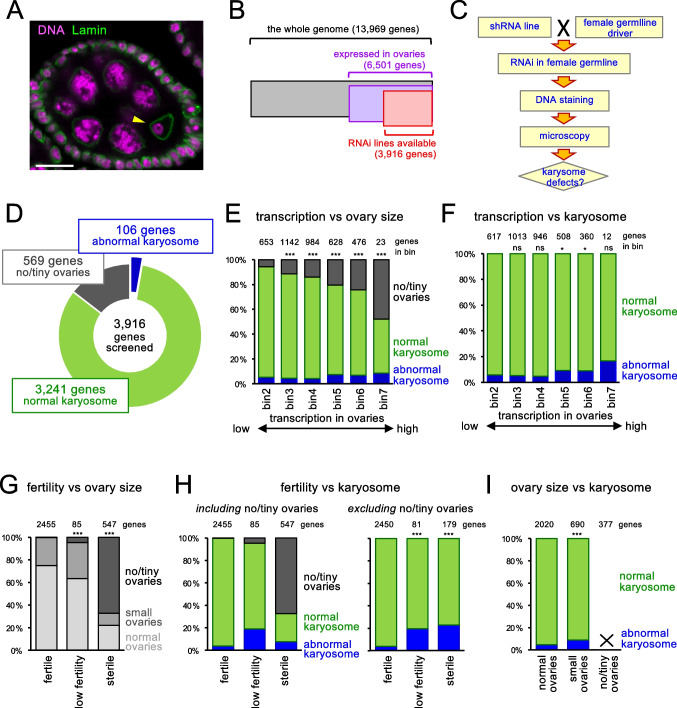
A large-scale screen identified 106 genes required for the integrity of the karyosome. **A** A stage-5 egg chamber from a control ovary stained for Lamin and DNA. The arrowhead indicates the karyosome, a compact spherical cluster of meiotic chromosomes, in the oocyte nucleus. Other nuclei belong to nurse cells and follicle cells. Bar = 10 μm. **B** The number of genes selected for the screen. **C** The karyosome screen workflow. **D** Summary results of the screen. Among 3916 genes screened, 106 genes showed reproducible karyosome abnormalities when they are silenced by RNAi, while 569 genes showed severe oogenesis defects which prevented examination of the karyosome. **E** The frequencies of genes with severe oogenesis defects and karyosome abnormalities when silenced, according to expression levels in ovaries. Bins 2, 3, 4, 5, 6, and 7 of the ovary expression level represent genes with 4–10, 11–25, 26–50, 51–100, 101–1000, > 1000 kb^−1^ million^−1^ from RNAseq (Mortazavi et al. 2008; Celniker et al. 2009). ***Significant differences (*p* < 0.001) in the frequency of genes with severe oogenesis defects in comparison to bin 2. **F** The frequencies of genes with karyosome abnormalities, excluding genes with severe oogenesis defects that prevented examination of karyosomes, according to different expression levels in ovaries. *Significant differences (*p* < 0.05) in the frequencies of genes with severe oogenesis defects in comparison to bin 2. ns no significant differences (*p* > 0.05). **G** The frequencies of genes resulting in different ovary sizes when silenced, in relation to female fertility. ***Significant differences (*p* < 0.001) in the frequencies of genes resulting in no or tiny ovaries in comparison to genes showing female fertility. **H** The frequencies of genes with karyosome abnormalities in relation to female fertility. The two graphs show the frequencies including or excluding genes resulting in no/tiny ovaries, which prevented examination of karyosomes. ***Significant differences (*p* < 0.001) in the frequencies of genes with abnormal karyosomes in comparison to genes showing female fertility. **I** The frequencies of genes with karyosome abnormalities according to their ovary size. No/tiny ovaries prevented examination of karyosomes. ***Significant differences (*p* < 0.001) in the frequencies of genes with abnormal karyosomes in comparison to genes with normal ovaries

In *Drosophila melanogaster* oocytes, the meiotic chromosomes together form a compact spherical cluster called the karyosome in prophase of the first meiotic division (Fig. 1A). To identify genes important for karyosome formation in *Drosophila melanogaster* oocytes, we carried out a genome-wide RNAi-based screen. By using RNAseq data from ovaries of mature 4-day-old females (Mortazavi et al. 2008; Celniker et al. 2009), we excluded genes expressed in ovaries at no/extremely low levels (0 reads per million; bin 0). Initially, we included genes expressing at a very low level (1–3 reads per million; bin 1), but due to a low frequency of hits during an early phase of the screen, these genes were also excluded from the screen. Among 13,969 genes in the *Drosophila melanogaster* genome, 6501 genes are expressed in ovaries at a low level or higher (≥ 4 reads per million; bin 2–7). Among these 6501 genes, 3916 genes had at least one available transgenic line suitable for RNAi in female germlines when we started the screen (Fig. 1B; Table S1).

For each of the 3916 genes, we tested one transgenic RNAi line from the TRiP collection (Ni et al. 2011). In these lines, expression of a short hairpin (sh) RNA against a target gene is controlled by the Gal4-responsive upstream activation sequence (UAS). Each RNAi line was crossed with flies expressing Gal4 in the germline under the *nanos* regulatory elements. This enables expression of shRNA starting from premeiotic stages in the female germline of the progeny. Dissected ovaries were fixed and stained for DNA (Fig. 1C). In the first round of the screen, we examined a small number of oocytes between oogenesis stages 3 and 9, which roughly correspond to zygotene to pachytene stages. In wild type or a negative control (RNAi of the *white* gene), the karyosome was spherical in most oocytes and slightly deformed in a small proportion of oocytes. If some karyosomes showed abnormal morphology, the shRNA lines were crossed with the same driver again to re-examine more karyosomes.

Among the 3916 genes, RNAi of 106 genes (2.7%) showed frequent karyosome defects (≥ 25% of 20 karyosomes examined in the second examination) (Fig. 1D). We could not examine the karyosome morphologies for 569 genes (14.5%) upon RNAi, as they showed severe oogenesis defects (no/tiny ovaries) that prevent examination. Our screen is likely to have missed many genes required for karyosome integrity due to unavailability of RNAi lines, inefficiency of RNAi, or severe oogenesis defects. Nevertheless, this is the first systematic, unbiased, large-scale identification of genes required for the karyosome integrity in oocytes. We focused our further studies on these 106 genes that showed frequent and reproducible karyosome defects.

### Genes important for fertility are enriched among the 106 genes required for the karyosome

To test a correlation between expression levels in ovaries and the karyosome or oogenesis defects, genes were grouped according to estimated amounts of mRNA from RNAseq (Mortazavi et al. 2008; Celniker et al. 2009). We found a strong correlation between the expression level in ovaries and severe oogenesis defects (Fig. 1E). The higher the expression level in ovaries is, the more likely RNAi showed severe oogenesis defects. Next, after excluding genes with severe oogenesis defects that prevent observation of karyosomes, we calculated the frequencies of genes with the abnormal karyosomes in relation to the expression levels in ovaries. Genes expressed at a higher level in ovaries are more likely to show abnormal karyosomes (Fig. 1F), although the correlation is not as strong as between expression level and severe oogenesis defects.

During the first round of the screen, we recorded the size of ovaries and tested fertility for most (~ 80%) of the genes. When ovaries are too small to examine the karyosome, they were recorded as “no or tiny ovaries.” When ovaries are substantially smaller than a control but large enough to examine the karyosome, they were recorded as “small ovaries.” Fertility was judged by production of larvae. When no larvae or a few larvae were observed, it was recorded as “sterile” or “low/reduced fertility,” respectively. Otherwise, it was recorded as “fertile.”

As expected, sterility is strongly correlated with no or very small ovary size (Fig. 1G). After excluding genes with severe oogenesis defects that prevent observation of karyosomes, we tested whether the karyosome defects are correlated with fertility or ovary size. Twenty-three percent of sterile lines and 20% of lines with reduced fertility showed abnormal karyosomes, while only 4% of fertile lines showed abnormal karyosomes (Fig. 1H). In contrast, 9% of lines with small ovaries showed abnormal karyosomes, while 5% of lines with normal sized ovaries showed abnormal karyosomes (Fig. 1I). Therefore, the integrity of the karyosome morphology is much more strongly correlated with fertility than ovary size which may represent overall ovary growth/health. Our results from a large-scale screen further highlights the importance of the karyosome for reproduction.

### The 106 genes are highly interconnected and include genes regulating chromatin, nuclear envelope, and actin

**Fig. 2:**
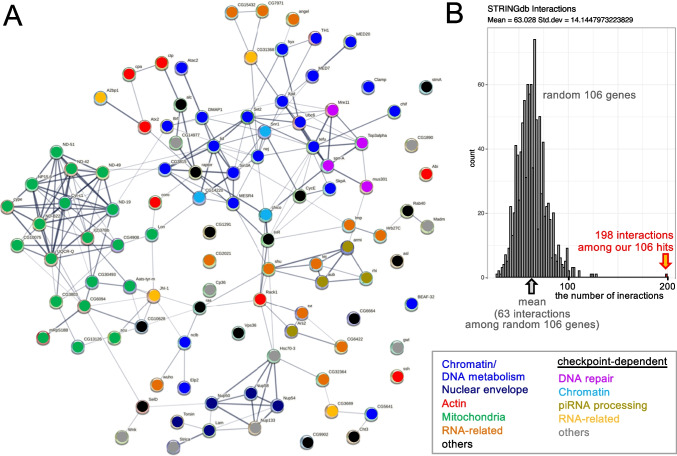
The 106 genes required for the karyosome are highly interconnected and include genes regulating chromatin, nuclear envelope, and actin. **A** The physical and/or functional interaction network among the 106 hits. Each node represents a gene identified in this screen and are coloured according to an associated key word indicated in the box. Each line represents a physical and/or functional interaction between two genes in the STRING database. **B** The numbers of interactions among random 106 genes. One thousand random sets of 106 genes were selected from 3356 genes examined in the screen, and the numbers of the physical and/or functional interactions were plotted. 198 interactions found among the 106 genes identified in the screen is much higher than expected from a random set of 106 genes

We concentrated our further studies on these 106 genes with reproducible and penetrant karyosome defects. We wondered how these 106 genes are related to each other and how they function together. Using STRING database (Szklarczyk et al. 2021), we found 198 known physical and/or functional interactions among the 106 genes (Fig. 2A). To test whether these interactions are more frequent than expected, we randomly selected 106 genes from the 3356 genes we have examined in the screen and counted how many known interactions are found among the random 106 genes. By repeating this 1000 times, we obtained a distribution of the number of interactions among the random 106 genes. This gave 63 interactions on average with a maximum of 128 interactions (Fig. 2B). Our 106 genes with karyosome abnormalities have > 3 times interactions than random sets of 106 genes. Therefore, the 106 genes identified in our screen are highly enriched in interactions. Among our 106 genes, only 15 genes are without known links to other genes, while all the others are directly or indirectly linked. This showed that we have identified a set of genes that are highly interconnected with each other.

Next, to find out what kind of proteins are encoded by these 106 genes, we compared gene ontology between these 106 genes with karyosome defects and the 3356 genes expressed in ovaries that we have screened (excluding genes with severe oogenesis defects). Gene ontologies over-represented among the 106 genes with karyosome defects include chromosome organisation, female gamete generation, nucleic acid metabolic process, and mitochondria-related terms. Over-representation of these terms are expected, with notable exceptions of mitochondria-related terms. This suggests that the screen was successful in identifying genes with potential roles in the meiotic chromosome organisation in oocytes. It also suggests that the substantial proportion of RNAi targeted the intended genes, which is consistent with a previous study using the same shRNA collection and driver that showed a very low frequency of off-target effects induced by shRNA (Sopko et al. 2014).

**Fig. 3:**
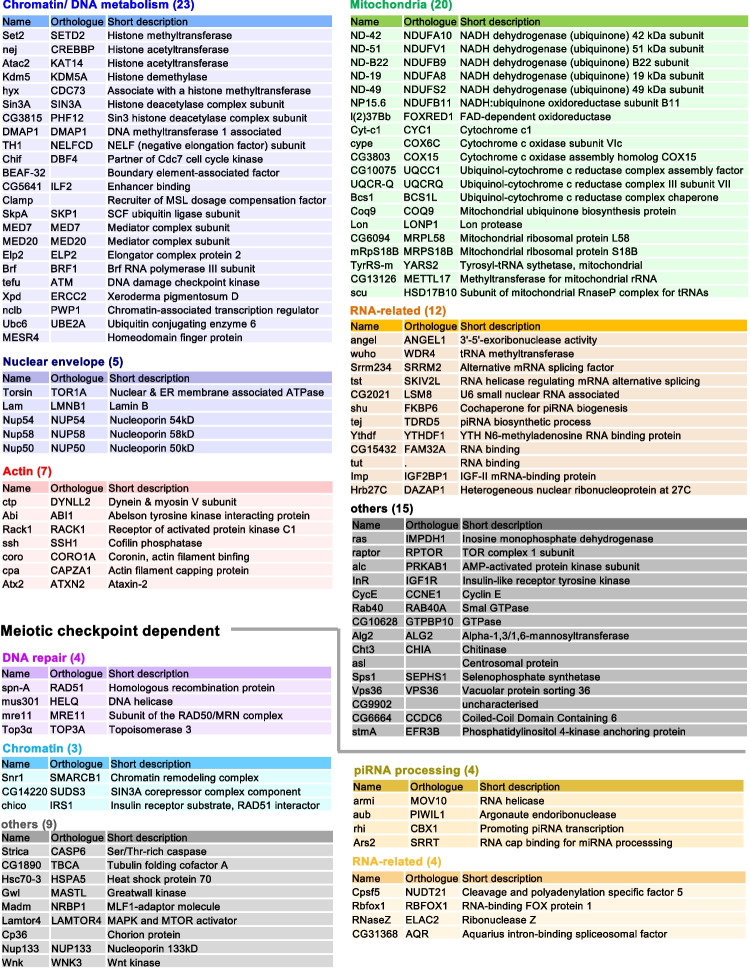
A list of the 106 genes important for the karyosome identified in the screen. The names of the 106 genes identified in the screen and their orthologue are shown with short summaries. They were grouped under common key words. Twenty-four genes are separately listed, as their karyosome defects caused by gene silencing are dependent on the meiotic checkpoint

Most genes (> 90%) identified in this screen are conserved in humans. Using information available on the genes and their orthologs, we manually grouped these 106 genes into categories (Fig. 3; Table S2). Some categories are related to functions or properties previously known to have links to the karyosomes, such as chromatin-related function (Zhaunova et al. 2016), nuclear envelope proteins (Breuer and Ohkura 2015), and actin regulators (Ilicheva et al. 2019). Others are related to functions or properties not previously implicated in the karyosome, such as mitochondrial proteins. Importantly, a significant proportion of the genes (14 out of 106 genes) have not been previously characterised in *Drosophila* and are only referred to as “CG” numbers. Therefore, our screen provides the first functional insights into these genes in *Drosophila*.

As expected, our screen identified chromatin proteins, chromatin-modifying enzymes, or proteins involved in DNA metabolism. It was previously reported that the histone demethylase Kdm5/Lid controls chromatin architecture in meiotic prophase I oocytes, although it does so independently of its catalytic activity (Zhaunova et al. 2016). Our screen results revealed that silencing of additional histone-modifying enzymes triggered karyosome defects. These genes include the histone methyltransferase Set2 (Stabell et al. 2007) and the histone acetyltransferases Nejire/CREBBP and Atac2 (Ogryzko et al. 1996; Suganuma et al. 2008). In addition, our screen also identified BEAF-32 (boundary element-associated factor of 32kD), which is known to regulate gene expression by modulating a higher-order chromatin structure (Gilbert et al. 2006).

Furthermore, our screen identified nuclear envelope proteins as expected. We have previously shown that the karyosome formation requires release of chromatin from the nuclear envelope and also the nuclear pores (Cullen et al. 2005; Lancaster et al. 2007; Breuer and Ohkura 2015). Attachment of chromatin to the nuclear envelope or pore complex is mediated by BAF or Nup155 and released by NHK-1 kinase or Nup62, respectively. Our screen identified Lamin and three further nuclear pore complex components, which could advance our understanding of how untethering of chromatin to the nuclear envelope or pore complex is regulated.

Interestingly, our screen also identified 7 proteins potentially regulating actin. Involvement of actin has previously been suggested for karyosome/karyosphere formation (Ilicheva et al. 2019; Maslova and Krasikova 2012). Actin is one of the main protein constituents of the karyosphere capsule in grass frogs (Ilicheva et al. 2019). In *Drosophila melanogaster*, karyosome formation was frequently delayed in mutants of *Scr64*, which encodes a tyrosine kinase regulating actin reorganisation during oogenesis (Djagaeva et al. 2005). Our results provide further evidence for the involvement of actin in karyosome formation and could serve a vital starting point for future mechanistic studies.

### Silencing of 24 genes results in karyosome defects mediated by meiotic recombination checkpoint activation

**Fig. 4:**
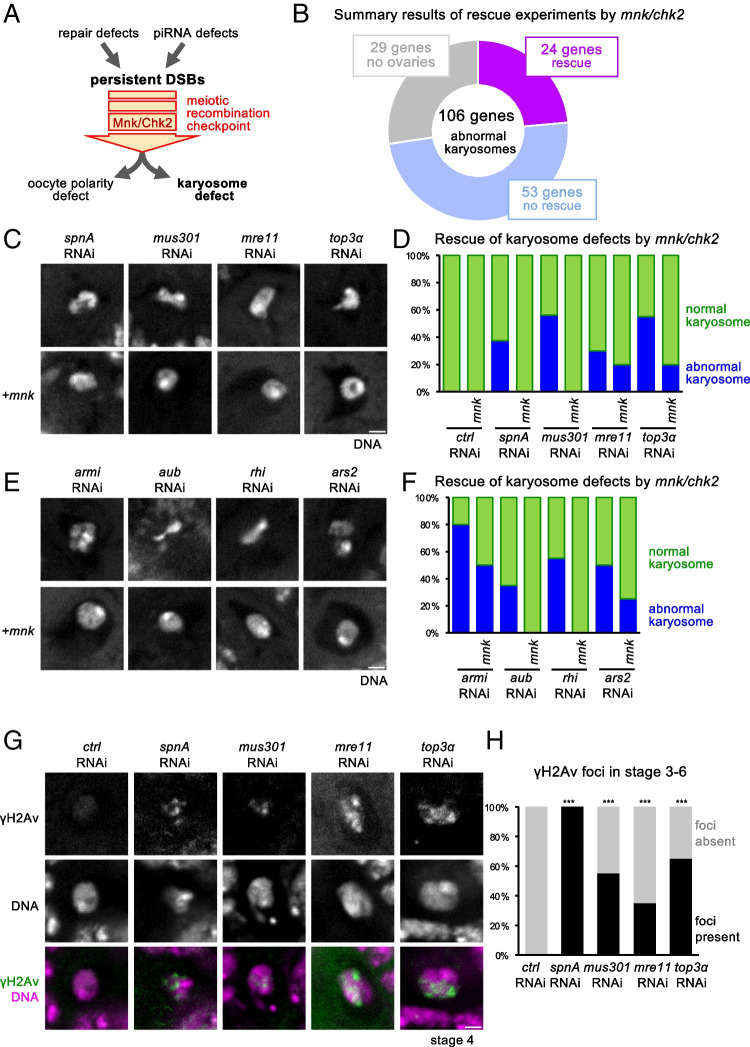
The karyosome defects of 24 genes are mediated by the meiotic recombination checkpoint. **A** A diagram of the meiotic recombination checkpoint that induces the karyosome defects in response to persistent DNA double strand breaks (DSBs). Persistent DSBs can be caused by failure of DNA repair or retrotransposition due to failure of piRNA-mediated silencing. **B** A summary result of rescue experiments by *mnk/chk2*. **C** The karyosome morphology in an oocyte in which a gene implicated in DNA repair was silenced by RNAi in the presence (+ *mnk*) or absence of a heterozygous *mnk/chk2* mutation. Bar = 2 μm. **D** The graph represents the frequencies of the karyosome morphologies in oocytes in which each gene involved in DNA double strand break (DSB) repair was silenced by RNAi in the presence (+ *mnk*) or absence of a heterozygous *mnk/chk2* mutation. **E** The karyosome morphology in an oocyte in which a gene implicated in piRNA processing was silenced by RNAi. Bar = 2 μm. **F** The graph represents the frequencies of the karyosome morphologies in an oocyte in which each gene involved in piRNA processing was silenced by RNAi. **G** Immunostaining of stage-4 oocyte using an antibody against γH2Av which marks DSBs. Bar = 2 μm. **H** The frequencies of stage 3–6 oocytes with γH2Av foci. ***Significant differences (*p* < 0.001) in the frequencies of oocytes with γH2Av foci in comparison to the control RNAi

During meiotic prophase I, DSBs (DNA double-strand breaks) are formed and repaired through recombination. In the face of persistent DSBs, the meiotic recombination checkpoint is activated and triggers defects in both karyosome formation and oocyte polarity (Morris and Lehmann 1999; Fig. 4A). Persistent DSBs can be generated by two alternative situations: a failure of DSB repair or a failure of suppressing retrotransposition by a piRNA-mediated mechanism. Therefore, the karyosome abnormalities of some of our hits may be induced by the meiotic checkpoint due to a defect in either DSB repair or piRNA processing.

To test whether the karyosome defects depend on the meiotic checkpoint, we suppressed the checkpoint by using a mutation in *mnk/chk2* encoding a key kinase essential for the functional checkpoint (Klattenhoff et al. 2007). We combined the *mnk/chk2* mutation with RNAi of 106 genes with reproducible and penetrant karyosome defects to see whether the *mnk/chk2* mutation rescues the karyosome defects. The *mnk/chk2* mutation, even when heterozygous over the wild-type allele, can suppress the karyosome defect in *spnA/rad51* RNAi, which is known to be essential for DSB repair (Fig. 4C, D). We found that, among the 106 genes with reproducible and penetrant karyosome defects, the defects of 24 genes (23%) were rescued by the *mnk/chk2* mutation fully or partially, and the defects of 53 genes (50%) were not rescued. The remaining 29 genes (27%) could not be determined mainly due to severe oogenesis defects when gene silencing and the *mnk/chk2* mutation were combined (Fig. 4B).

As expected, these 24 genes with checkpoint-dependent karyosome defects include genes (or their orthologues) already known to be required for DSB repair (*spnA*, *mus301/spnC*, *mre11*, *top3α*; Fig. 4C, D) or piRNA processing (*armi*, *aub*, *rhi*, *ars2*; Fig. 4E, F) in some experimental systems. As neither *top3α* nor *mre11* have been previously reported to be involved in karyosome formation, meiotic DSB repair or checkpoint dependency in *Drosophila* oocytes, we aimed to confirm that silencing of these genes results in persistent DSBs in *Drosophila* oocytes. In wild type, DSBs were formed in region 2a and fully repaired by region 3 in the germarium. We immunostained the ovaries expressing shRNA against a gene with an antibody recognizing a phosphorylated H2A variant (γH2Av) that marks DSBs (Fig. 4G, H). In control RNAi oocytes, γH2Av foci were only observed at early stages of meiosis in the germarium, but not in later-stage oocytes (stage 3 or later, which roughly corresponds to zygotene to pachytene stages), showing timely DSB repair. In contrast, γH2Av foci were still observed in oocytes at later stages when *top3α* or *mre11* was silenced, similarly to *spnA* or *mus301/spnC*. These results suggest that *top3α* and *mre11* are important for efficient repair of meiotic DSBs in *Drosophila* oocytes.

### Gene silencing of mitochondrial proteins leads to distinct karyosome defects

**Fig. 5:**
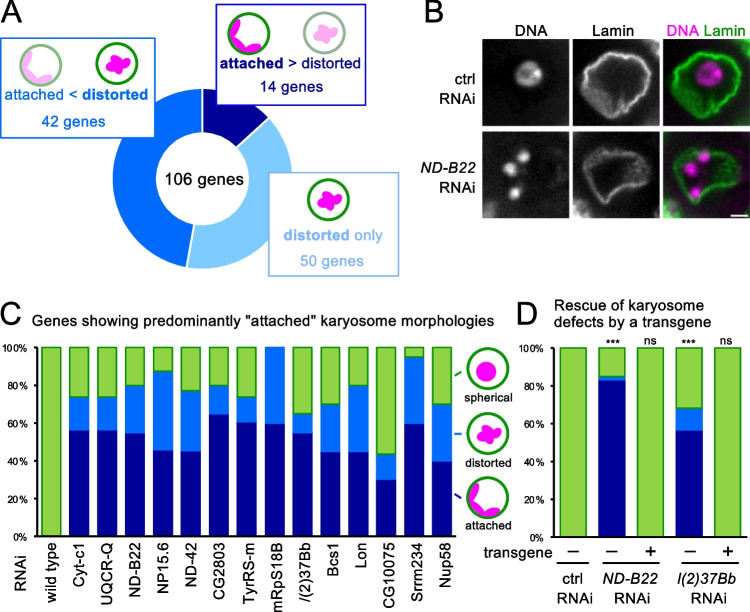
Mitochondrial dysfunction leads to distinct karyosome defects. **A** The numbers of genes showing predominantly attached, predominantly distorted, and only distorted karyosome morphologies upon silencing. **B** Immunostained nuclei of stage-6 oocytes expressing control and *ND-B22* shRNAs using a Lamin antibody and the DNA probe DAPI. RNAi of *ND-B22* gene encoding a mitochondria protein predominantly shows an “attached” karyosome morphology, in which meiotic chromosomes, often three masses, are located in proximity to the nuclear envelope. Bar = 2 μm. **C** Genes showing predominantly attached karyosome morphologies upon silencing. Twelve out of 14 genes in this category encode proteins with roles in mitochondria. **D** Rescue of the karyosome defects of *ND-B22* or *l(2)37Bb* RNAi by an RNAi-resistant wild-type *ND-B22* or *l(2)37Bb* transgene, respectively. ***Significant differences (*p* < 0.001) in the frequencies of oocytes with abnormal karyosomes in comparison to the control RNAi. ns; no significant differences (*p* > 0.05)

The 106 genes with reproducible and penetrant karyosome defects were further analyzed for the karyosome morphology. We classified abnormal karyosome morphologies and distribution into two categories (Fig. 5A). The normal karyosome morphology is largely spherical, and chromosomes are in one mass commonly away from the nuclear periphery, or appear to contact the nuclear periphery only at small areas. The first category of abnormal morphologies (called “distortion”) includes a chromosome mass (or masses) whose overall shape is far from spherical, but largely away from the nuclear periphery. The second one (called “attachment”) includes karyosomes in which the overall shape of the chromosome mass (or masses) is far from spherical and is located very close to the nuclear periphery.

Among the 106 genes with reproducible and penetrant karyosome defects, silencing of 14 genes (13%) predominantly showed attachment morphology more than the distortion morphology. Silencing of 42 genes (40%) showed both abnormal morphologies but the distortion morphology is predominant. Silencing of the remaining 50 genes (47%) showed only distortion morphology (Fig. 5A).

Interestingly, 12 out of 14 genes predominantly showing attachment morphology encode proteins with a known or a likely role in mitochondria (Fig. 5B, C). In addition to the similarity of the karyosome morphologies, the karyosome defects in all of the examined genes that predominantly show attachment morphology are independent from the meiotic checkpoint (Figure S1; Table S2).

To confirm that the karyosome defects are due to depletion of the mitochondrial proteins and not off-target effects, we selected two of the genes (*ND-B22* and *l(2)37Bb)* encoding mitochondrial proteins that are part of the electron transport chain for rescue experiments. An RNAi-resistant wild-type *ND-B22* or *l(2)37Bb* transgene was generated and expressed in ovaries together with shRNA against *ND-B22* or *l(2)37Bb*, respectively. The karyosome defects were fully rescued by expression of the wild-type transgenes (Fig. 5D). This demonstrated that silencing of *ND-B22* and *l(2)37Bb* is responsible for the observed karyosome defects.

### Gene silencing of a mitochondrial protein has multiple phenotypic consequences in female meiosis

**Fig. 6:**
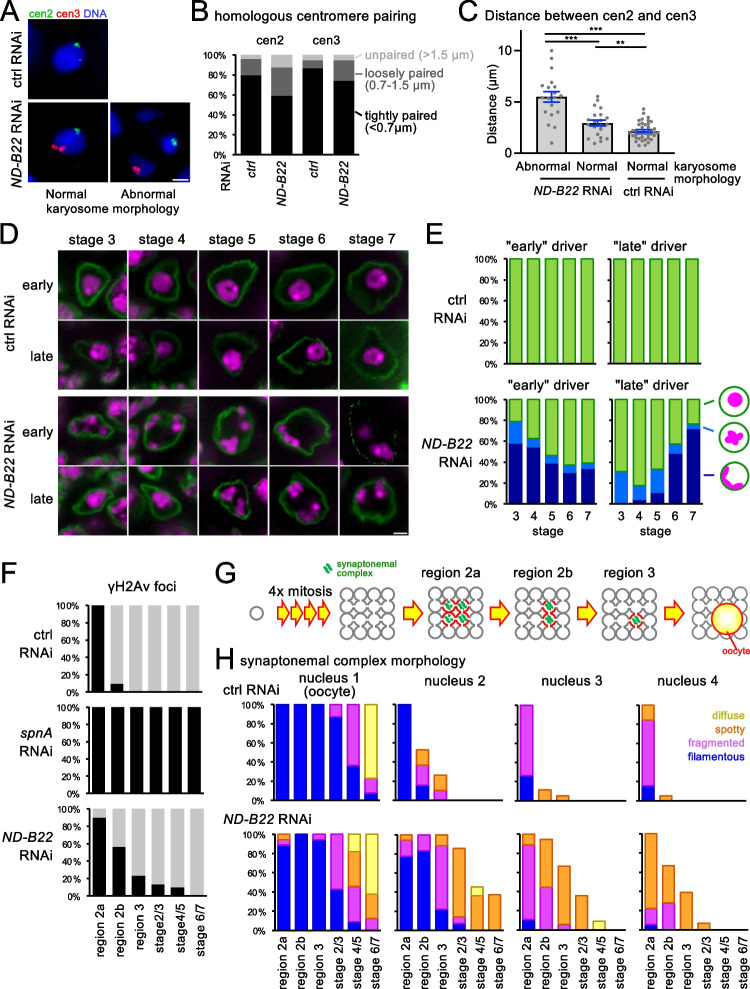
Silencing of ND-B22 encoding a mitochondrial protein has multiple phenotypic consequences in female meiosis. **A** Fluorescence in situ hybridisation of the karyosome using peri-centromeric satellites of chromosomes 2 and 3 (cen2 and cen3) in oocytes with control RNAi and *ND-B22* RNAi. **B** The distances between cen2 signals or between cen3 signals. They are categorized into three groups based on the distances. **C** The distance between cen2 and cen3 signals. The error bars represent the standard errors of the means. ***p < 0.001. **p < 0.01. **D** The karyosome morphologies in different stages of oocytes expressing shRNA against *ND-B22* using an early or late driver. Ovaries were immunostained using a Lamin antibody and DAPI. Bar = 2 μm. **E** Frequencies of the karyosome morphologies in different stages of oocytes expressing shRNA against *ND-B22* using an “early” (nos-Gal4) or “late” (mat-α-tubulin) driver. **F** The frequency of meiotic cells containing foci of γH2Av, a DSB marker, in various stages of *ND-B22* RNAi ovaries. **G** A progenitor cell undergoes four mitotic divisions to generate 16 interconnected cells. The synaptonemal complex is formed in four cells and completes disassembling in two of the cells first and then in the third cells. The remaining cell (the oocyte) gradually disassembles the synaptonemal complex in later stages. **H** Morphologies of the synaptonemal complex (C(3)G) in four meiotic nuclei in various stages of oogenesis. The nucleus with the most well formed synaptonemal complex is assigned as nucleus 1, and the nucleus with second, third, and fourth most well formed are assigned as nucleus 2, 3, and 4, respectively

We further characterized the karyosome and other defects in meiotic processes, induced by silencing of *ND-B22*, which encodes a component of the electron transport chain. In the wild-type karyosome, sister-centromeres are cohesed together and homologous centromeres then paired (Dernburg et al. 1996). To test whether centromere cohesion and pairing are affected by knockdown of mitochondrial proteins, we visualized centromeres by fluorescence in situ hybridization probed with pericentromeric satellites from chromosomes 2 and 3 (Fig. 6A). In over 80% of control RNAi oocytes, one signal or a pair of closely located signals (< 0.7 μm) were observed for each probe, showing cohesion of sister-centromeres and tight pairing of homologous centromeres (Fig. 6B). In *ND-B22* RNAi, one signal or a pair of closely located signals were still observed for each probe in most oocytes, but loosely paired signals (0.7–1.5 μm) were more often observed than in a control RNAi (Fig. 6B). These results suggest that *ND-B22* RNAi does not affect sister-centromere cohesion but slightly loosens pairing of homologous centromeres. To test whether relative locations of non-homologous centromeres are affected, distances between signals from peri-centromeric satellites of chromosome 2 and chromosome 3 were measured (Fig. 6C). The measurement revealed that the distance between these two signals was significantly larger than in the control RNAi. This is the case even in *ND-B22* RNAi oocytes that still maintain a spherical karyosome morphology. It suggests that the internal organization of the karyosome is disrupted even within the spherical karyosome morphology in *ND-B22* RNAi oocytes.

To test at which stage the karyosome becomes abnormal, the karyosome morphology was observed stage by stage. When shRNA was expressed using the GAL4-induced promoter and a routinely used GAL4 driver (driven by *nanos* regulatory elements), the karyosome defects were observed from stage 3, at which point the karyosome is formed in the wild type (Fig. 6D, E). This indicates that normal mitochondria function is important for karyosome formation.

To test whether mitochondria function is important also for maintenance of the karyosome, we first allowed the karyosome to form before depleting the mitochondria protein sufficiently by delaying the start of expression of shRNA. We used Gal4 driven by the maternal α-tubulin promoter that starts expressing Gal4 later than *nanos* regulatory elements we routinely used (Matthews et al. 1993). At stages 3 and 4, spherical karyosomes were formed and maintained in most of oocytes. However, by stage 7, meiotic chromosomes were attached to the nuclear envelope in most oocytes (Fig. 6D, E). This showed that mitochondria function is important for maintenance of the karyosomes as well as formation.

During the recombination process in meiotic prophase, DSBs are formed and repaired. To test whether DSBs are formed and repaired at the right timing, ovaries were immunostained with an antibody against phosphorylated H2Av (γH2Av) that marks DSBs (Fig. 6F). In control, DSBs were formed in region 2a and fully repaired by region 3. Next, we examined RNAi of *spnA*/*rad51* that is essential for DSB repair. As reported before in a *spnA*/*rad51* mutant, DSBs in RNAi oocytes fully persisted until late stages of the oogenesis (at least stage 6/7). In contrast, *ND-B22* RNAi oocytes showed that DSBs were formed and started disappearing at the expected timings, but the disappearance was significantly slower than control RNAi. Eighty to 90% of oocytes successfully repaired DSBs by stage 2 or 3, while 10–20% still retained DSBs. However, eventually nearly all the DSBs were repaired. This delay of DSB repair potentially activates the meiotic checkpoint pathway to prevent the formation of spherical karyosomes. However, the data described above (Table S2; Figure S1) showed that inactivation of the checkpoint failed to rescue the karyosome defects caused by *ND-B22* RNAi. Therefore, there must be another cause of the karyosome defects other than just the delay of DSB repair.

Next, we examined the dynamics of the synaptonemal complex during meiotic progression. In wild type (Fig. 6G), one progenitor cell undergoes four mitotic divisions to form 16 interconnected cells. The synaptonemal complex is first assembled as filamentous structures in the nuclei of four cells in region 2a of each germarium. In two of them, including the future oocyte, the synaptonemal complex is fully assembled, while it is only partially assembled in the other two cells. Three of the cells, excluding the future oocyte, start disassembling the synaptonemal complex, and the disassembly is first completed in two of the cells by region 2b and then in the third cell by region 3. This leaves the synaptonemal complex only in the oocyte. Disassembly in the oocyte starts at stage 3 and gradually progresses (Page and Hawley 2001).

To test whether dynamics of the synaptonemal complex is affected, immunostaining was carried out using an antibody against the transverse filament protein C(3)G. In control RNAi, we found similar dynamics as observed in wild type. In *ND-B22* RNAi, more cells contained the synaptonemal complex in the germarium in comparison to the same region in the control, although the number of cells with the synaptonemal complex did not exceed 4 in each region. To estimate the dynamics of the synaptonemal complex for each cell, the C(3)G pattern of each cell in a region (each cluster) was separately recorded (Fig. 6H). It showed that the dynamics of the synaptonemal complex in the most persistent cell was similar to wild-type oocytes. Disassembly of the synaptonemal complex in the other three meiotic cells was slower than the equivalent cells in wild type. This demonstrated that the dynamics of the synaptonemal complex showed little change in oocytes, although the disassembly was delayed in other cells that initially form the synaptonemal complex.

### Karyosome defects related to mitochondrial dysfunction are not due to apoptosis

**Fig. 7:**
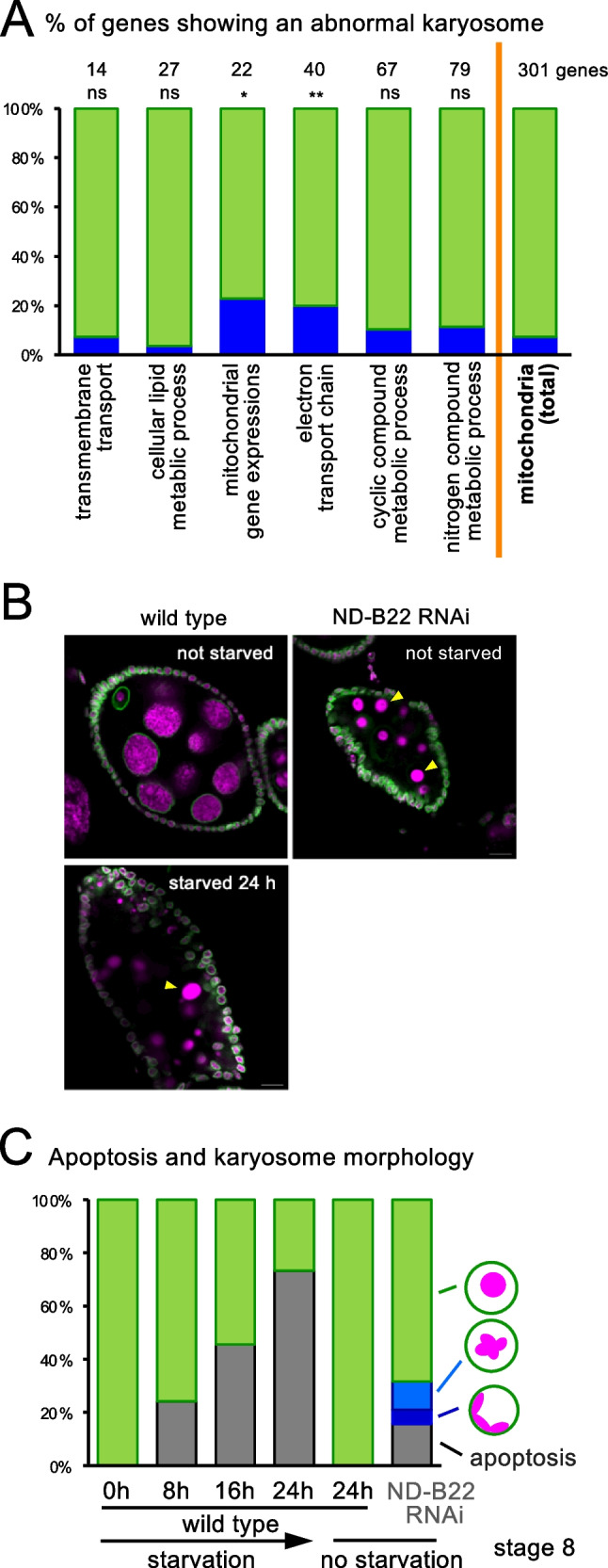
Karyosome defects caused by mitochondria dysfunction is not mediated by apoptosis. **A** Frequencies of genes with different mitochondrial functions that show karyosome defects upon silencing. ** and *Significant differences (*p* < 0.01 and 0.05, respectively) in the frequency of genes with abnormal karyosomes, in comparison to the other genes with mitochondrial functions. **B** Immunostaining of apoptotic stage-8 egg chambers in wild-type oocytes after 24 h of starvation, along with healthy stage-8 egg chambers without starvation. All chromatin in *ND-B22* RNAi and wild type starved for 24 h shows abnormal morphology associated with apoptosis in this figure, except in the follicle cells. Some examples are indicated by the arrowheads. Bar = 10 μm. **C** Frequencies of apoptosis and karyosome morphologies in stage-8 egg chambers in wild-type oocytes after different lengths of starvation, along with in *ND-B22* RNAi

To gain insights into which mitochondrial function is important for the integrity of the karyosome, we analyzed the results of our screen to determine the proportion of genes with each mitochondrial function that gave karyosome defects when silenced (Fig. 7A). A significantly higher proportion of genes with electron transport chain function gave karyosome defects when silenced, in comparison to the other genes with mitochondria functions. In addition, genes involved in gene expression of the mitochondrial genome are significantly more likely to give karyosome defects. This also points to the electron transport chain, as all 13 proteins encoded by the mitochondrial genome have roles in the electron transport chain (Wolstenholme and Clary 1985). In contrast, genes involved in various metabolic processes were not significantly more likely to give karyosome defects. Although it is impossible to make a firm conclusion, this may suggest that dysfunction of the electron transport chain might cause the karyosome defects.

Next, we wish to understand how mitochondrial dysfunction leads to a change in chromatin organisation in the oocyte nucleus. One possibility is that mitochondrial dysfunction may trigger apoptosis which in turn leads to an abnormal karyosome. This is an attractive hypothesis, as apoptosis is known to link mitochondria to nuclear events. It has been shown that apoptotic cells in ovaries undergo a series of characteristic changes of chromatin morphologies (Etchegaray et al. 2012). In particular, the nuclear envelope of germline cells is disintegrated in the course of cell death. The chromatin is ultimately fragmented to form amorphous balls of various sizes.

To test whether mitochondrial dysfunction may trigger apoptosis, we first examined whether apoptosis is triggered by silencing of *ND-B22*, which encodes an essential mitochondrial protein. DNA staining of ovaries with *ND-B22* RNAi showed chromatin morphologies characteristic to apoptosis in a low frequency of egg chambers (Fig. 7B, C). Most oocytes with karyosome abnormalities were found in egg chambers with a healthy appearance. Although the observed abnormal karyosome morphologies caused by silencing of *ND-B22* are distinct from the morphology in apoptotic oocytes, it is still possible that the observed abnormal karyosome morphology is an intermediate step of apoptosis. To test this possibility further, we induced apoptosis in wild-type ovaries without RNAi by starving flies. It is known that starving wild-type female adults triggers apoptosis in oocytes and nurse cells in stage-8 egg chambers (Terashima and Bownes 2004). When we starved female adults for various lengths of time (8–24 h), stage-8 oocytes (or egg chambers) showed chromatin morphologies characteristic of apoptosis (Fig. 7B, C). The longer the starvation lasted, the more frequently apoptosis was observed, reaching 70% after 24 h of starvation. However, in any oocytes after any length of starvation, we did not observe karyosome morphologies similar to the ones observed in RNAi of genes with mitochondria functions.

In conclusion, although mitochondrial dysfunction causes a low frequency of apoptosis, apoptosis is unlikely to be the cause of the characteristic karyosome defects seen upon mitochondrial dysfunction.

## Discussion

We carried out a large-scale screen of *Drosophila melanogaster* oocytes to identify genes with a function in the karyosome formation. The karyosome screen comprised 3916 candidate genes, covering 60% of all genes expressed in ovaries except the ones with extremely or very low expression. It yielded 106 genes with strong and reproducible karyosome defect upon knockdown.

This screen is a highly systematic approach aimed at the identification of genes that would not be picked up from a pre-selected and limited set of candidate genes. Unlike a selective approach, it minimized a bias towards genes with known functions or domains likely to be involved in karyosome formation. Indeed, this screen included 953 previously uncharacterized genes. Among them, 14 showed karyosome abnormalities upon knockdown and our screen thus provided the first functional information on these genes.

Nevertheless, our screen will have missed some genes important for karyosome formation. First of all, we did not examine genes not expressed or only expressed at very low levels in ovaries (0–3 reads per million by RNAseq; Graveley et al. 2011). Just over a half (7468 out of 13,969) of *Drosophila melanogaster* protein-coding genes fall into this category. Initially, we included genes expressed at very low levels (1–3 reads per million), but due to a low frequency of hits during an early phase of the screen, we excluded these genes. Secondly, among 6501 genes expressed in ovaries at the levels of ≥ 4 reads per million, 2585 genes did not have transgenic RNAi lines suitable for gene silencing in the germline (Ni et al. 2011) at the start of the screen. Thirdly, expression of shRNA may not deplete the gene product sufficiently to result in phenotypic consequences. It was roughly estimated that about a half of shRNA in the same collection using the same driver do not sufficiently deplete the gene product in eggs (Sopko et al. 2014). In the future, our screen can be refined using a second shRNA to minimise these false negatives. Finally, silencing of 569 genes out of 3916 genes in our screen resulted in severe oogenesis defects, preventing any examination of the karyosome. Some of these may have roles in karyosome formation as well as oogenesis. Our screen hits are likely to be more biased towards genes with functions specific to the karyosome. A different driver that directs either weaker or later expression can be used in future studies to overcome this issue.

A further question to be considered is the false discovery rate (FDR) among the 106 genes identified in this screen. This is likely to result largely from off-target effects of shRNAs. A previous study using this shRNA collection and the same driver suggested the FDR is very low. It would be difficult to directly apply this very low FDR to our screen, as the aims and assay in our screen were different from those in their screen (Sopko et al. 2014). Nevertheless, it will probably also be low in our screen, as the majority of our hits have a function and/or property potentially related to the karyosome. Our hits are highly interconnected with much more known functional and/or physical interactions than expected from a random set of genes. They include genes with known or possible functions previously implicated in the karyosome formation, such as regulating chromatin (Zhaunova et al. 2016), nuclear envelope (Breuer and Ohkura 2015), and actin (Ilicheva et al. 2019).

It is known that persistent DSBs, due to failures in DSB repair or piRNA processing (which suppresses retrotransposition), activates the meiotic recombination checkpoint, which induces karyosome defects as well as oocyte polarity defects (Abdu et al. 2002). We found the karyosome defects of 24 of our hits can be rescued by silencing of the meiotic checkpoint. Indeed, they include 4 genes with known function in DNA repair and we confirmed that silencing of these genes results in oocytes with DSBs even in late stages. Furthermore, 4 genes known to be involved in piRNA processing have been identified. The remaining 16 genes are potentially novel genes that have roles in DSB repair or piRNA processing in oocytes.

The most unexpected finding is that our screen has identified many genes with a function in mitochondria. A rescue experiment using an RNAi-resistant wild-type *ND-B22* or *l(2)37Bb* gene confirmed that this is unlikely to be due to an off-target effect. Furthermore, we found other defects in meiotic progression, including delays in synaptonemal complex disassembly in cells not destined to become the oocyte. DSB repair is slightly delayed but suppressing the meiotic recombination checkpoint did not rescue the karyosome defects. Therefore, the checkpoint activation is not the main cause of the karyosome defect induced by gene silencing of mitochondrial proteins.

How do mitochondrial defects lead to nuclear defects? Mitochondria are known to play an important role in triggering apoptosis, which alters the organization of the cell structure and function including chromatin structure in the nucleus (Jeong and Seol 2008). Indeed, we found that silencing of nuclear-encoded mitochondrial genes results in apoptosis in some egg chambers. However, when apoptosis was induced by starvation in wild-type ovaries, karyosome defects typical of mitochondrial dysfunction were not observed. This suggests that mitochondrial dysfunction independently triggers apoptosis and karyosome defects.

What links mitochondria defects to the karyosome defects? The main function of mitochondria is ATP production, and our results indeed suggest the karyosome integrity is sensitive to gene silencing of electron transport chain components. Therefore, a reduction of the ATP concentration in oocytes may cause the karyosome defect. As many cellular processes including phosphorylation require ATP, a failure to carry out any one or more ATP-dependent processes may be responsible for the karyosome defect. In addition, iron-sulphur clusters are generated by mitochondria (Lill and Mühlenhoff 2008). Defects in generation of iron-sulphur clusters in mitochondria are known to result in genome instability (Veatch et al. 2009). Some DNA metabolic enzymes in the nucleus require iron-sulphur clusters, including DNA helicases (Rudolf et al. 2006) and DNA glycosylases (Alseth et al. 1999). Therefore, a reduced level of iron-sulphur clusters may decrease the activity of some of the DNA metabolic enzymes important for the karyosome integrity in the nucleus. Our screen has identified Xpd/ERCC2, a DNA helicase, known to require iron-sulphur clusters (Liu et al. 2008; Pugh et al. 2008). It would be of future interest to examine whether this enzyme links mitochondria defects to the karyosome defects.

An alternative possibility is that mitochondria dysfunction is sensed by a “stress” responsive signaling pathway to trigger the alteration of the karyosome morphology. This is not exclusive to other possibilities, as an altered concentration of ATP or other molecules caused by mitochondrial dysfunction may be sensed by a signalling pathway. We showed that activation of apoptosis in wild type does not induce the karyosome defects. Other known pathways responsive to stress, such as hypoxia, may be worth investigating in the future.

Errors in female meiosis I are widely recognised as the major cause of miscarriage and intellectual disability in humans even though little is known about their molecular origins. Formation of meiotic chromatin clusters in oocytes has been proposed to be important for faithful chromosome segregation. Emerging evidence suggests that mitochondria affect all aspects of mammalian reproduction, since they are essential for oocyte maturation, fertilization, and embryonic development. In this context, our results showing that mitochondrial dysfunction triggers karyosome defects provides molecular insights into the link between mitochondrial diseases, chromatin arrangements, and infertility.

## Materials and methods

### Drosophila genetics

Flies were maintained at 25 °C on cornmeal medium following standard techniques of fly handling (Ashburner et al. 2005). For gene silencing in ovaries, the respective TRiP RNAi fly stocks were obtained from the Bloomington Drosophila Stock Center (BDSC) and crossed with a specific *GAL4* driver line (Ni et al. 2011). The two *GAL4* driver lines used in this study were MVD1 (*P[GAL4::VP16-nos.UTR]CG6325*^*MVD*1^; BDSC4937) and V37 (*P[Matα-Tubulin67C-Gal4::VP16] V37*; BDSC7063). Meiotic checkpoint suppression was obtained by a heterozygous mutation, *mnk*^*p6*^ (Klattenhoff et al. 2007). For control RNAi, progeny from a cross between *white* RNAi (BDSC 35,573) and the same *GAL4* driver line as other RNAi was used.

### Cytological techniques

Oogenesis in the female progeny was stimulated with dry yeast in the presence of males at 25 °C for 2 days.

#### DAPI staining

Ovaries from five females were dissected in Robb’s medium (100 mM HEPES pH 7.4; 40 mM potassium acetate; 10 mM glucose; 1 mM CaCl_2_; 55 mM sodium acetate; 100 mM sucrose; 1.2 mM MgCl_2_) and ovarioles fixed for 10 min in 100 µl fixation solution (200 mM Cacodylate pH 7.2; 80 mM potassium acetate pH 7.5; 20 mM EGTA; 200 mM sucrose; 20 mM sodium acetate pH 5.2) at room temperature. DNA was counterstained with 100 µl DAPI solution (0.4 µg/ml; Sigma) overnight. The following day, ovarioles were transferred into mounting medium (2.5% propyl gallate w/v; 85.5% glycerol) and mounted onto slides.

#### Nuclear envelope staining

Ovaries were dissected and ovarioles fixed as described above (see DAPI staining). Following fixation, ovarioles were blocked in 200 µl blocking solution for 2 h at room temperature. Ovarioles were transferred into 100 µl of PBSTx (PBS + 0.1% Triton X-100) containing mouse anti-Lamin primary antibody (1/100; ADL67.10; Developmental Studies Hybridoma Bank) and left rotating at room temperature overnight. The following day, ovarioles were washed three times in PBSTx for 10 min each and transferred into 100 µl secondary antibody solution (1/250 in PBSTx; Alexa Fluor 488 AffiniPure Donkey anti-mouse IgG; Jackson Immuno Research) including DAPI (0.4 µg/ml; Sigma). Incubation was allowed at room temperature overnight as before. The following day, ovarioles were washed (3 × 10 min in PBSTx) and mounted onto slides.

#### Synaptonemal complex staining

Ovaries were dissected in cold PBS and ovarioles fixed for 20 min. Ovarioles were washed (3 × 10 min in PBSTx) before blocking. Incubation with the primary antibody solution, including rat anti-C(3)G (1/100 in PBSTx; Zhaunova et al. 2016) and rabbit anti-γH2Av (1/50 in PBSTx; Lancaster et al. 2010), was allowed at room temperature overnight. The following day, ovarioles were washed (3 × 10 min in PBSTx) and transferred into secondary antibody solution, including donkey anti-rat Cy3 (1/250 in PBSTx, Jackson) and goat anti-rabbit Alexa 488 (1/250 in PBSTx, Thermo Fisher Scientific). DNA was counterstained with DAPI (0.4 µg/ml; Sigma) and incubation allowed for 4 h at room temperature. Ovarioles were washed (3 × 10 min in PBSTx) and mounted onto slides.

#### Examination of the synaptonemal complex

The C(3)G localization pattern in meiotic prophase was classified into four patterns: “filamentous” (showing relatively long filamentous structures that represent a fully assembled synaptonemal complex), “fragmented” (showing very short filamentous structures that represent the synaptonemal complex starting to disassemble or in the process of assembling), “spotty” (showing a small number of strong C(3)G foci in the nuclei), and “diffuse” (showing a C(3)G signal that is diffused evenly in the nucleoplasm, representing complete disassembly). The identification of germarium regions was based exclusively on morphological criteria. Notably, region 3 of the germarium is connected to a stage-2 egg chamber by a linear monolayer of stalk cells. In contrast, region 2b and region 3 of the germarium are connected by multilayer of stalk cells. In region 2a of the germarium, there were some uncertainties in defining which four cells with the synaptonemal complex belong to the same germline cyst, when clusters of these cells were close together.

### Imaging techniques

Immunostained oocytes were imaged with a confocal scan head LSM800 attached to an Axiovert 200 M (Zeiss) using PlanApoChromat objective lens (63x/1.4 numerical aperture) with Immersol 518F oil (Zeiss). Z-sections were captured with 0.5-µm interval, 512 × 512-pixel/zoom 2 (~ 0.1 µm/pixel). The maximum intensity projection of several *Z*-planes or one single Z-plane representing the region of interest is shown in the figures. One Z-plane representing the region of interest is shown in the figures. Images were exported as tagged image file (TIFF) and edited using ImageJ. Contrast and brightness were adjusted uniformly across the field using ImageJ.

### Screening process

#### Choice of genes to screen

This screen includes all genes in the *Drosophila melanogaster* genome meeting two following criteria. (1) The expression level in ovaries is low or higher (≥ 4 reads per million per kilobase) judged by RNAseq (Graveley et al 2011). (2) At least one RNAi line based on the VALIUM20 or VALIUM22 vector (Hu et al. 2017) is available from the Bloomington Drosophila Stock Center (BDSC) (up-to-date information in December 2018 obtained from https://bdsc.indiana.edu/stocks/rnai/rnai_all.html).

#### Fly crosses

To silence each candidate gene in the female germline cells, three males from the respective RNAi stock were crossed with three virgin females from the *nos-GAL4-VP16* MVD1 driver line. Fly food used in crosses was supplemented with some dry yeast. The vials were incubated for 7 days at 25 °C before removing the parents. Ten days after crossing, non-balanced female progeny was selected for maturation and dissection. To stimulate oogenesis, five females were incubated in the presence of three males and dry yeast on fly food for 2–3 days at 25 °C prior to dissection.

The workflow for each candidate gene comprised four steps: (1) RNAi-mediated knockdown of the candidate gene in ovaries, (2) stimulation of oogenesis in females, (3) dissection of ovaries and DNA staining, and (4) examination of the karyosome using confocal microscopy. The ovary size of females was noted during dissection and classified as either normal, small, or no/tiny in comparison to wild-type females. In the initial round of the screen, the karyosome was examined in about six oocytes between oogenesis stage 3 and stage 9 per candidate gene. The karyosome could not be examined when the candidate gene knockdown resulted in tiny or completely underdeveloped ovaries. The karyosome was classified as “normal” when its shape was spherical, slightly deformed, or slightly elongated. The karyosome was classified as “abnormal” when the chromatin formed a strongly distorted mass or discontinuous chromatin masses. To assess how candidate gene knockdown affects karyosome morphology, both the frequency and severity of observed karyosome defects were considered. All candidate genes with dissectible ovaries were classified based on karyosome morphology. The genes were considered to have “abnormal” karyosomes, when at least three out of six examined oocytes showed abnormal karyosome morphology. In addition, when one or two oocytes showed an abnormal karyosome morphology, the genes may be considered to have “abnormal” karyosomes depending on the overall impression across the karyosomes. These genes with “abnormal” karyosomes were selected for the second round of the screen for validation of the karyosome defects.

#### Validation of screen results

The workflow in the second round of the screen is exactly the same as described above, except that 20–29 oocytes were examined per candidate gene. One hundred six genes showed karyosome abnormalities in 25% or more of all examined oocytes in the second round. These 106 genes were considered to show reproducible karyosome defects and therefore studied further. In all 106 genes with karyosome defects, there were two major types of karyosome defects: chromatin attachment to the nuclear envelope and chromatin distortion in the nucleus.

#### Meiotic checkpoint examination

The dependence of karyosome defects on meiotic checkpoint activation was tested in the 106 genes with frequent and reproducible karyosome defects. shRNA was expressed using the *nos-GAL4* driver (MVD1) in the presence or absence of a heterozygous mutation of the central checkpoint kinase *mnk*^*p6*^ (Klattenhoff et al. 2007). The karyosome defects were considered to depend on the meiotic checkpoint, when 75% or fewer of oocytes showed karyosome defects in the presence of the *mnk* mutation in comparison to the absence of the *mnk* mutation. In total, 10–29 oocytes per gene were examined for suppression by the meiotic recombination checkpoint.

### Fertility assay

To test how silencing of a candidate gene in the germline affects fertility, a test vial was set up 10 days after crossing the respective RNAi fly stock with the *nos-GAL4-VP16* MVD1 driver. In this vial, five females and three males from the non-balanced F1 progeny were incubated for 15 days at 25 °C before recording fertility. The F1 progeny was considered to be fertile when the number of progeny in the F2 generation was comparable to wild-type flies. When the number of F2 progeny was substantially reduced, the F1 progeny was considered to have reduced fertility. The F1 progeny was considered sterile in the complete absence of larvae or pupae.

### Fluorescent in situ hybridization (FISH)

#### Probe generation

Probes targeting the pericentromeres of chromosome 3 (cen3) and chromosome 2 (cen2) were generated from oligonucleotides labelled with fluorescent dUTPs. (AACAC)_6_ labeled with Alexa Fluor 488–5-dUTP (Thermo Fisher) was used for the cen2 probe, and an equal mixture of (CCCGTACTGGT)_2_ and (CCCGTACTCGGT)_2_ labeled with Alexa Fluor 546 14-dUTP (Thermo Fisher) was used for the cen3 probe. The reaction mix contains 1 × Terminal Transferase Buffer (Promega), 1.5 U/µl Terminal Deoxynucleotidyl Transferase (TdT; Promega), 0.8 mM dTTP, and 5 µM oligonucleotides (cen2 or cen3, respectively). The reaction mix was incubated at 37 °C for 1 h and the enzyme inactivated at 70 °C for 10 min. After cooling down at room temperature for 5 min, oligonucleotides were purified using a MiniQuick column (Sigma-Aldrich) as follows. The column matrix (G-50) was resuspended, and the column centrifuged (3550 rpm, 2 min). After the reaction mix was applied to the centre of the column bed, the column was centrifuged (3550 rpm, 4 min) and the recovered eluate stored at − 20 °C.

#### Hybridization

After females had been in the presence of males and dry yeast supplement at 25 °C for 2 days, their ovaries were dissected in Robb’s medium. Entire ovaries were fixed for 4 min and ovarioles separated in 2xSSCT. Ovarioles were transferred into a 0.5 ml PCR tube and washed three times in 2xSSCT for 10 min each. Ovarioles were incubated in three SSCT solutions with increasing formamide concentration for 10 min each (2xSSCT 20%/40%/50% formamide) and in fresh 2xSSCT 50% formamide at 37 °C for 30 min. The probe solution (250 ng of each probe in 1.1 × hybridization buffer) was heated to 91 °C for 10 min and frozen on dry ice. Immediately after thawing, the probe solution was added to ovarioles, and the samples were incubated at 91 °C for 2 min. The temperature was then reduced to 37 °C to allow annealing overnight. The following day, ovarioles were washed three times in 2xSSCT/50% formamide for 10 min before reducing the formamide concentration in two sequential steps (2xSSCT 40%/20% formamide). Ovarioles were washed three times in 2xSSCT (no formamide) and two times in PBSTx before staining with DAPI.

#### Measurements

The distance between homologous centromeres was measured when two separate dots can be distinguished. When the homologous centromeres appeared as one single dot, a distance of 0 µm was recorded for further analysis. Centromeres were considered to be tightly paired (0–0.7 µm), loosely paired (0.7–1.5 µm), or unpaired (≥ 1.5 µm) based on the measured distance. The distance between non-homologous centromeres in 3D was calculated from two measurements (*x*/*y* and *z*) in the maximum intensity projection. When two dots were observed for homologous centromeres, the measurements were made from the estimated mid-point between them.

### Statistical analysis

The Fisher’s exact test calculator for a 2 × 2 contingency table or *t* test calculator for two independent means (two-tailed) provided by Social Sciences Statistics was used to test for significance at *p* < 0.05 (www.socscistatistics.com).

### Apoptosis time course

The starvation of *Drosophila melanogaster* females via deprivation of a suitable protein source triggers apoptosis at stage 8 of oogenesis (Terashima and Bownes 2004). To examine a possible co-occurrence of apoptosis and karyosome defects in wild-type (*w*^*1118*^) females, they were starved on sucrose solution for an extended period of time. In this time course experiment, only recently eclosed females (within 16 h) were included. The young females were first matured on full cornmeal medium including yeast supplement for 46 h. The females were then kept in the presence of filter paper soaked with 10% sucrose solution for 8 h, 16 h, or 24 h prior to dissection and DAPI staining as described above. In oogenesis, the degradation process in apoptotic egg chambers can be followed using DNA staining (Etchegaray et al. 2012). To distinguish between non-apoptotic (phase 0–phase 1) and apoptotic (phase 2–phase 5) egg chambers at stage 8 of oogenesis, the classification published by Etchegaray et al. (2012) was followed. The stage of apoptotic egg chambers was thereby deduced from surrounding healthy egg chambers.

### Bioinformatics

The following basic settings of STRING (https://string-db.org, version 11.5) were chosen when generating the protein interaction network or determining the number of physical and/or functional interactions among the 106 genes identified in the screen. These settings are full STRING network for “network type,” confidence for “meaning of network edges,” all for “active interaction sources,” and medium confidence (0.400) for “minimum required interaction score.” To simulate the expected number of protein interactions amongst genes identified in the screen, 1000 sets of 106 random genes drawn from the 3356 candidate genes examined in the screen.

For Gene Ontology (GO) terms and gene groups, FlyBase (Release 6.32) was used to generate a list of all *Drosophila melanogaster* genes associated with the GO term “Cellular component: mitochondrion” (GO:0,005,739). This list contains 917 genes, of which 344 were used in the screen. STRING (https://string-db.org) was used to manually identify the six following Gene Ontology groups (biological processes) that have 10 or more genes for further examination: GO:0,034,641 “Cellular nitrogen compound metabolic process,” GO:1,901,360 “Organic cyclic compound metabolic process,” GO:0,022,900 “Electron transport chain,” GO:0,140,053 “Mitochondrial gene expression,” GO:0,044,255 “Cellular lipid metabolic process,” and GO:1,990,542 “Mitochondrial transmembrane transport.”

## Supplementary Information

Below is the link to the electronic supplementary material.

Figure S1. The karyosome defects upon gene silencing of mitochondrial proteins are independent from the meiotic recombination checkpoint. The graph represents the frequencies of the karyosome morphologies in oocytes in which each gene for mitochondrial proteins was silenced by RNAi in the presence (mnk) or absence of a heterozygous mnk/chk2 mutation. X without a bar represents no/tiny ovaries that prevent examination of the karyosome morphology. (PDF 20 KB)

Table S1. A summary of 3916 genes used in the screen. This table includes FlyBase ID, gene symbol, TRiP RNAi line, expression level in ovaries, summary results of the first round of the screen, summary results of the second round of the screen, fertility and ovary size. (XLSX 214 KB)

Table S2. A summary of the 106 genes identified in the screen. This table includes results of the second round of the screen, gene function, karyosome morphology and dependency on the meiotic recombination checkpoint. (XLSX 51 KB)

## Data Availability

The screen data is included as supplementary materials. The materials are available on request.
