# Supplementary figures and images for: A large-scale RNAi screen reveals that mitochondrial function is important for meiotic chromosome organization in oocytes
Source: Chromosoma. 2023 Jan 17;132(1):1–18. doi: 10.1007/s00412-023-00784-9 (PMC9981535; doi:10.1007/s00412-023-00784-9)

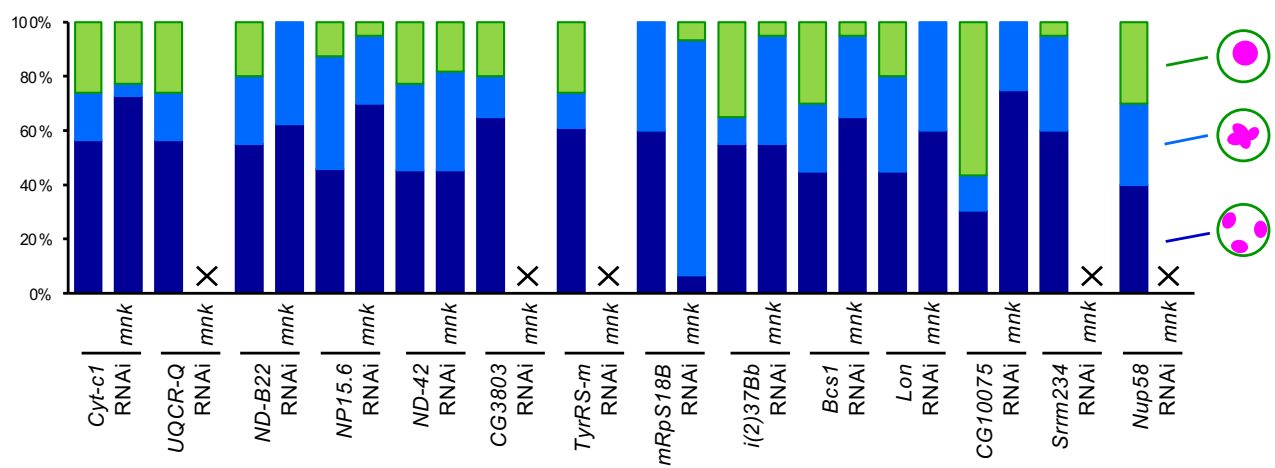

Figure S1

Supplement: Supplementary file 1 — Figure S1. The karyosome defects upon gene silencing of mitochondrial proteins are independent from the meiotic recombination checkpoint. The graph represents the frequencies of the karyosome morphologies in oocytes in which each gene for mitochondrial proteins was silenced by RNAi in the presence (mnk) or absence of a heterozygous mnk/chk2 mutation. X without a bar represents no/tiny ovaries that prevent examination of the karyosome morphology. (PDF 20 KB) [file 412_2023_784_MOESM1_ESM.pdf]
